# New York Academy of Medicine

**Published:** 1857-09

**Authors:** 


### ECLECTIC DEPARTMENT,

### AND SPIRIT OF THE MEDICAL PERIODICAL PRESS

---

### *New York Academy of Medicine.—Puerperal Fever.*

(Continued from Page 114)

Prof. Clark said: I will make a few remarks on the subject asking your indulgence for the reason already given. It will be remembered that the pathology of this disease occupied a considerable portion of the attention of the Academy, on a former occasion. I then stated that the fever was in its nature *inflammatory*, that it is a fever with demonstrable lesions in every case. I also stated my conviction that those cases described by Gooch, Simpson, and others, as without lesion were cases of pyæmia, and that pyæmia has its source in the inflammation of the inner surface of the uterus; and the facility with which the uterine sinuses could convey the pus into the system, was shown. Where a patient has died of puerperal fever, supposed to be without lesions, I had been able to demonstrate the existence of this pus, and I have not been able to demonstrate it in cases where patients have died of puerperal convulsions. In addition to the views then given, I would state that where inflammatory fibrinous exudation has been found on the inner surface of the uterus, without pus, pus may still be the result of the same inflammation as it extends to terminal uterine sinuses.

In two cases, lately, I have found secondary deposits of pus in distant parts of the body, the results of the purulent contamination, and yet the secretion was not pus, but was fibrinous on the inner surface of the uterus.

We have lately had a form of puerperal fever at Bellevue Hospital, with the pathology of which I have not before been familiar, and which, I have reason to think, has not been so thoroughly studied by the profession as that of other forms. Its prominent lesion is inflammation of the inner surface of the uterus, with evidences of general purulent contamination; and the disease is unusually protracted. The patients have lived, in several cases, from ten to thirty days; and the *post-mortem* examinations have demonstrated the existence of secondary purulent deposits, and pus on the inner surface of the uterus, and in its veins or lymphatics.

This form of the disease is more insidious in its approach—is often devoid of the symptoms that mark the onset of the ordinary attacks of puerperal fever, and is more allied to typhoid fever. I can best illustrate this by giving the history of a case, drawn up for me by Dr. McEwen, of the Hospital corps.

CASE I.—Clara Byrne, native of England, æt. 26 years; was delivered, April 25, the duration of her labor being one hour and a half; was a passenger on board the *Cornelius Grinnell*, arrived in this country after a voyage of five weeks, April 21st, 1857; she suffered from sea sickness during the whole voyage.

April 26. Was admitted to the hospital to be delivered; half an hour after being admitted, her labor began, and she was delivered of a healthy child; she complained of nothing till May 1.

May 1. Had a slight diarrhœa; stated that she had been deaf for a few days past, and that the deafness was gradually increasing; complained of slight headache and shooting neuralgic pains in the left supra and infra-orbital nerves.

Ordered—lotion of chloroform and aconite, for neuralgia, and gr. ss. of morphine to procure sleep.

May 2. Neuralgia had disappeared; headache had disappeared; complained of general feeling of malaise; lochia were normal.

May 3. Headache worse; slight epistaxis from left nostril; half dozen dry cups to the neck relieved the headache.

May 4. Diarrhœa continues, worse in the evening; tongue furred and white in the centre; pulse 80.

Ordered—castor oil and laudanum, gtts. 40.

May 5. Diarrhœa not as bad; the feeling of general weakness had increased; experienced slight chilly sensation, most marked toward evening. (This recurrence of the chilliness toward evening will be found to be a marked symptom of the case.) Symptoms continued the same up to

May 9. Diarrhœa continues; slight white fur on the tongue, and a disposition to become dry; lochia stopped.

Repeated castor oil and laudanum; port wine every hour.

At 6, P. M., had a severe chill, lasting about fifteen minutes, with increased feeling of general weakness.

9, P. M. Face flushed; tongue rather dry; pulse 100, and intermitting; headache very severe; bronchitic râles heard over the whole chest; a slight murmur heard at the base of the heart; systolic, and not conducted to the vessels.

May 10. Pulse 128; weakness increased; deafness marked. (Thus it will be seen that the symptoms have come on gradually; there has been no marked period of invasion.) Transferred from the lying-in-wards to ward 23.

5, P. M. Pulse 120; skin hot and perspiring; lochia ceased; diarrhœa slight; two discharges; tympanitis slight; *no tenderness on pressure over abdomen or over uterus;* no mammary secretion; tongue dry, and slightly coated brown; face flushed.

May 11. Pulse 92; symptoms the same, except that the diarrhœa had ceased.

5, P. M. Pulse 116; skin still hot and perspiring.

May 12, 9, A. M. Pulse 96; deafness not so marked; other symptoms the same.

3, P. M. Had a well-marked chill, followed by increased heat of skin, and profuse perspiration.

5, P. M. Pulse 125; face flushed; skin hot; perspiring profusely; delirious.

10, P. M. Skin hot; perspiring profusely.

May 13, 8, A. M. Pulse 84; skin cool and moist; slight tympanitis; *no tenderness on pressure;* tongue brown and dry; no mammary secretion; no lochial discharge; deafness much diminished.

4, P. M. Had a slight chill, followed by increased heat of skin, and profuse perspiration.

5½, P. M. Pulse 116; still perspiring; somowhat delirious.

7, P. M. Pulse 124; still perspiring; somewhat delirious.

12, M. Pulse 128; diarrhœa slight: three or four liquid discharges; passes her urine involuntarily; still perspiring.

May 14, 5, A. M. Pulse 140; much worse; skin cool and moist; no mammary secretion; no diarrhœa; no tenderness on pressure; tympanitis diminished; tongue very dry and brown; no lochial discharge.

9, A. M. Perspiration profuse.

11, A. M. Had a slight chill.

10, P. M. Had a slight chill.

May 15, 9. A. M. Pulse 108; skin cool and moist; no tympanitis; *no tenderness;* no lochial discharge; no mammary secretion; no diarrhœa; tongue brown and dry; still passes her urine involuntarily.

10, A. M. Had a well-marked chill.

6, P. M. Pulse 130; face flushed; perspiration profuse.

8, P. M. Symptoms the same.

May 16, 9, A. M. Pulse 160; had a chill at seven; skin hot and perspiring; face flushed; no lochia; no tenderness; no mammary secretion; no diarrhœa; no incontinence of urine.

6, P. M. Pulse 170; other symptoms the same.

Died at half past 9, P. M.

*Autopsy eighteen hours after death.* External appearances: no erythematous spots; slight hypostatic congestion of body; tympanitis scarcely noticeable, making inconsiderable elevation of the umbilical region; vagina dry, but having a whitish, varnished look, as if secretion had dried upon it; watery secretion of breasts.

*Lungs.* Healthy, except apex of left, were there were a few tubercles and a small cavity; no fluid in the pleural cavities.

*Heart.* Natural size; pericardium and heart structure healthy; slight thickening of aortic valves; others healthy; half an ounce of transparent serum in the pericardium.

*Liver.* Full size, rather soft, and somewhat fatty at points.

*Gall Bladder.* Filled with nearly healthy bile.

*Spleen.* Twice the natural size, and much softened.

*Kidneys.* Inferior anterior portion of the right, showing somewhat the appearance of fatty degeneration; left presenting somewhat more the color and appearance of fatty degeneration than the right; not strongly marked in either.

*Stomach.* Membrane of a pinkish hue; markedly mammillated in pyloric half; red with punctate ecchymosis in cardiac half; entire membrane somewhat thickened; gastric glands somewhat enlarged, and of a red color.

*Duodenum.* Gastric half markedly mammillated; second half somewhat hyperæmic.

*Jejunum.* Somewhat more vascular than usual; at the lower portion, solitary glands enlarged and distinct (white, as if filled with fluid pus); Peyer's patches begin to be visible, mapped out into sections by irregular whitish bars, running across them variously; passing down ileum, Peyer's patches become more and more distinct, slightly elevated, of a grayish color, but none ulcerated, except the last at the ilio-cœcal valve, where an ulcer existed of the size of a half a dime, looking more like the result of tuberculous than of typhoid disease. (This is an important point. In the opinion of the young gentlemen of the staff this was a simple penetrating ulcer. I state this, because the question might arise whether this ulcer was not of the typhoid fever character.) Solitary glands, enlarged, whitish, and elevated.

*Large Intestines.* Solitary glands very distinct; membrane otherwise of healthy appearance; mesenteric glands, connected with ileum, moderately enlarged, and red.

*Uterus.* Blood, fluid, or imperfectly coagulated, in all the large vessels; length, including the neck, four inches and a quarter; width of body, laid open, three inches and a half; on dissecting its connections with left side of pelvis, pus flowed in considerable quantity; pus apparently infiltrated into the tissue anterior to the neck of the bladder, and behind symphisis pubis; several vessels, either lymphatic or venous, in the substance of the organ, contained some purulent fluid; from the seat of the purulent deposit, in appendages of left side, a lymphatic vessel or vein was traced upward to the top of fundus, enlarged, and filled with pus; three or four small abscesses in broad ligament of the left side, one communicating with the vessel just referred to; in two others, no such communication could be found; the veins of uterus, in neighborhood of placental attachment, nearly all filled with coagula.

The enlarged sac just outside the valvular mouth in one instance enlarged and filled with pus. One or two small accumulations of purulent matter in tissue of uterus just without its inner surface.

*Inner Surface.* Itself covered with a somewhat flocculent fibrinous exudation; stained of a deep brown, almost of a black, color, by a fluid of a creamy consistence and of the same hue, which covered the whole inner surface. The quantity of this fluid retained in the uterus from four to six ounces; it contained pus.

*Neck.* Same color as the body; tissue somewhat softened; remains of placenta still attached.

*Fallopian Tubes.* Contained pus; very red.

*Ovaries.* Nothing noticeable.

Here is a case in which all the symptoms on which reliance usually is placed in the diagnosis of puerperal fever, were absent at the commencement. No chill, no headache, no pain in abdomen, no rapid rise in the pulse. In fine, there was no well-marked period of invasion, such as we have been taught to expect in that disease. The first symptom that is noticed is the diarrhæa, and that occurred on the fourth day. In ordinary peritonitis, the bowels are constipated, but in this class of cases the bowels either are loose or easily moved by medicines. The whole disease is marked by its *gradual* progress; the characteristic point is the recurrence of the chill and the long perspiration that followed the chill, or of free perspiration without chills. So regular was this in the case just read, that I expected, and almost invariably found, my patient in a perspiration when I made my daily visit.

I wish to call particular attention to the condition of the solitary glands of intestines in this case. They were swollen, filled with a milky fluid apparently, and stood out on the surface of the mucous membrane, in certain parts, like pustules, as they often do in small-pox and cholera. I have been induced, lately, to attach importance to this lesion and its connections with pyæmia, having met with it frequently during the epidemic here described; and in some cases of purulent infection unconnected with the puerperal state. Dr. Heywood will recollect such a case; one that we examined together a few days ago. The Peyerian patches also participate in the diseased action in some of the cases.

Thus, the analogies of this case all ally it to common purulent phlebitis. Such it undoubtedly would have been considered, had it occurred in any other than the puerperal state, or even in this state, perhaps, if it had occurred alone. But its associations are no less important the disease itself. It occurred at the end of a series of eleven cases, four of which had the peritoneal inflammatory lesions, and six appeared to suffer mainly from uterine phlebitis and pyæmia. Nine of the other ten cases occurred between the second and the twelfth of April. These cases have been recorded for me by Drs. McEwen and Buist, of the hospital staff. [They were too long to be read before the Academy, but the following synopsis of them has been furnished to the reporter, with remarks.]

Case II.—Jane C.; delivered March 27; time of attack uncertain, within three or four days, insidious; no chill, or marked rigors; no general abdominal tenderness; circumscribed tenderness in hypogastric region; prostration; pulse 120 and upward, without force or volume; green vomiting.

April 6. Skin hot; face occasionally flushed; no constipation; no secretion of milk; tympanitis inconsiderable; death on the tenth day after delivery, April 7, and on the fourth or fifth, probably, of the disease.

*Lesions.* Lymph, pus, and serum on intestines and uterus, and in the cavity of the abdomen. Internal surface of uterus stained to an appreciable depth of dark brown or pale pink hue, covered by a reddish brown, pasty effusion. consisting of blood, plastic cells, and fibres, and epithelium; veins and lymphatic vessels did not appear to contain pus; stomach and mucous membrane pale, thickened, and mammilated; tympanitis, even after death, inconsiderable.

*Remarks* The women who are attacked by puerperal fever are sent to the general fever wards as soon as the disease can be recognized; and it often happens that the lying-in wards and the fever wards are in charge of different attending physicians. This was the case in April, when this and the other cases here reported, occurred. This case was not transferred, and Dr. Clark did not see it until time of the *post-mortem* examination. The evidence of peritonitis, at the autopsy, were of the utmost marked character; yet, the physician who treated the case is confident that there was no pain in the abdomen, or tenderness, except as described above.

CASE III.—Magdalena M., delivered March 28, after an easy labor.

April 8, eleven days after, was found with a pulse of 140, hot skin, and slight tenderness over uterus. Pulse fell to 72, and tenderness disappeared after a few hours' use of sulph. morphiæ ($\frac{1}{2}$ gr. every hour.) Same symptoms repeated after four days, with tympanitis; no chill; free perspirations first and second days after apparent renewal of attack, slight afterward; abdominal tenderness on second day; *no pain complained of;* vomiting fourth day; bloody vaginal discharge; pulse 116 to 160; death twenty-first day after delivery, tenth after first symptoms, and sixth after renewal of attack. Transferred to fever wards April 12, 7, P. M.

*Post-mortem appearances.* Tympanitis very considerable; peritoneum covered with plastic effusion; eight ounces of milky serum in abdominal cavity; inner surface of uterus covered by a red diffluent exudation, containing pus; mouths of sinuses patulous; inner layer of fibres stained reddish; no pus seen in veins or lymphatics; pus in vesicles of right ovary; mucous membrane of stomach injected and thickened; pyloric portion mammillated; intestines ecchymotic in spots; Peyer's patches elevated; incipient gangrene (?) of left lung.

CASE IV. Hannah C., delivered, April 21, 1857, at 8, P. M., after an ordinary labor; membranes somewhat adherent; pulse, immediately after, 110, next morning, 118; at 6, P. M., tenderness on right side of uterus; pulse 140, and, at midnight, 160.

April 23. Tenderness over whole of abdomen; tympanitis marked; vaginal discharge, dark, bloody; tongue dry; no milk; pulse 140 to 180; decubitus dorsal; legs drawn up; in afternoon, sweated profusely for some hours; died at 4, A. M., April 24, two days and eight hours after delivery, and thirty-four hours after disease was recognized; no chill; patient was supposed to have albuminuria; transferred, sixteen hours before death.

*Post-mortem appearances.* Tympanitis considerable; shreds of lymph on peritoneal surface, here and there; a few ounces of turbid serum in its cavity; capillary injection noticeable, but not extreme; evidences of inflammation only slight; pus in the external layer of uterine veins and lymphatic vessels, also, in those of the broad ligaments; inner surface covered with a somewhat firm, mammillated layer of yellowish exudation; color of inner fibres of uterus, bright pink; ovaries inflamed; some vesicles enlarged, and contained whitish fluid; punctate ecchymosis of mucous membrane of stomach; in some of Peyer's patches, vesicles enlarged, containing a purulent-looking fluid; injection of the valvulæ conniventes; fibrinous exudation at the vulva.

Case V. Harriet Q., delivered, April 10, after a labor of twenty-eight hours' duration, by forceps; pulse excited from the time of the labor, 110 and upward, quick and irritable; skin hot, and respiration frequent; second day, pulse 120; pain on pressure over hypogastric region, and extending upward; tympanitis; vaginal discharge, second day, of dark color; third day, offensive; tympanitis became extreme; tenderness mostly confined to hypogastric region; no pain; free perspirations, from the fourth day onward; dry, brown tongue, and involuntary evacuations on fifth day, and after; delirious on eighth day; pulse 100 to 136; suppurative inflammation of parotid on sixth day; chill on seventh; death on tenth day after delivery; transferred, April 12, 7, P. M.

*Lesions.* Tympanitis extreme; no inflammatory effusions of any kind on intestines, or in abdominal cavity; pus and fibrine on the inner surface of uterus; muco-purulent fluid in fallopian tubes; pus in right parotid gland, and in tissues, just anterior to vagina; local congestions of mucous membrane of intestines; pyloric extremity of stomach mammillated and thickened; pure pus discharged from vagina; kidneys dotted over with small, yellowish, white spots, which, on microscopic examination by Dr. Draper and self, separately, were found to consist of pus.

Case VI. Ann W., delivered, April 7, 1857, after an ordinary labor.

April 9. Colicky pains; bowels constipated for past five days; pulse 96; cheeks flushed; no chill; (bowels relieved.)

April 10. Some tympanitis; countenance anxious; pulse 112; skin hot; face flushed; afternoon, green vomiting.

11th. Free perspirations at night.

12th. Vaginal discharge ceased, then returned, of dark color, not offensive; tympanitis continues; slight tenderness over uterus; mind wandering.

13th. Vaginal discharge, profuse and offensive.

From 13th to 23d. Frequent and free perspirations; occasional green vomiting; tympanitis remaining, but not extreme; bowels, at first, slow, afterward, too free; mammary secretion scanty; occasional delirium; skin at times dry and hot; vaginal discharge becoming light yellow; tongue alternately brown and dry, then moist; pulse, restrained by medicine, 88 to 128; tenderness in hypogastric region remaining, but not markedly increasing; strength diminishing.

April 23. Tympanitis nearly gone; tenderness remaining.

24th. Tympanitis returned; death on the afternoon of the 25th, after fifteen or sixteen days' illness; transferred, April 10.

*Lesions.* Large peritoneal abscesses filling pelvis, and rising as high as the level of superior spinous processes of ilium, shut in by adhesions of the intestines; no general peritonitis; abscesses in both broad ligaments, one of which had opened into peritoneal cavity, and communicated with large abscess; others just ready to open; fibrinous and purulent effusion on inner surface of uterus; three sloughy ulcers in rectum, opposite large abscess; mucous membrane of stomach purplish, thickened, and most of it mammillated; follicles of jejunum distinct.

The patient who is the subject of the following report, was lying in the same ward, and was seized at the same time, with two others, whose disease terminated in five and nine days, respectively, one, No. 2, of this series, presenting the usual peritoneal lesions; the other, No. 11, was supposed to have had metro-peritonitis, but the opinion was not verified by an autopsy. This circumstance, as well as the interest with which the case was watched during its progress by physicians not attached to the hospital, may, perhaps, render a synopsis unsatisfactory, and justify the unusual length of the record. The history was kept by Dr. Buist.

Case VII. Mary Ann Fay, æt. 17 years; of rather delicate constitution; was admitted into the lying-in wards of Bellevue Hospital, March 11, being in her first pregnancy. On April 2, at 10 o'clock, A. M., she gave birth to full-grown living child, after a natural labor of some twelve hours' duration; the placenta was expelled in ten or fifteen minutes after, and the uterus contracted firmly. During the evening of the same day she complained of slight pain, and tenderness on pressure, in the right iliac region. The day following, the lochia appeared, and were reported in all respects natural; tenderness remained the same; no chill had occurred, but the pulse was frequent, and she did not appear well; on the 4th, the pain and tenderness had almost subsided, wet cups and a turpentine stupe having been applied the day previous; heat of skin and irritability of pulse became quite marked; lochial discharge was somewhat offensive; she was slightly nauseated, but did not vomit; there was only a moderate secretion of milk; and she showed much langor and feebleness; during the 5th, 6th, 7th, and 8th, she continued very much in the same condition, with a pulse averaging 130; a flushed face; respiration about normal; the lochia entirely ceasing. On the evening of the 8th, being considered a case of puerperal fever, she was removed from the lying-in department to another ward; up to this time, she had taken but a very small amount of opiates, with a few doses of calomel a day or two after delivery. (When the woman was sent to the fever ward for treatment, and from the day before, when Dr. Clark first saw her, her condition was regarded as hopeless, and no active treatment was adopted; she was looked upon as one already fatally pyæmic, but, surviving thirty-six hours from her transfer, she was put upon quinine and brandy, etc., which were continued till the secondary meningitis began.)

April 8. Quite feeble and languid, but in no pain; bowels confined, and excessively tympanitic; tongue dry, and coated slightly; cheeks flushed; pulse 140 to 144; respiration 15 to 20 in the minute.

April 9. Feels comfortable and disposed to dose; gets one ounce brandy every two hours, and beef tea; has eaten an egg; no vomiting.

9, P. M. Sweating profusely; tongue more moist; sleeps occasionally; bowels confined; is in no pain whatever.

April 10. Sleeps well toward morning; pulse very weak; tongue dry and tremulous.

7, P. M. Sleeping; has passed urine in bed; pulse 150 to 156; stimulants to be increased, with the addition of sulph. quinine.

April 11. Bowels moved at 7, A. M.; stool dark and semi-fluid; pulse, through the day, 152 to 136; respiration 16 to 18.

April 12. Skin hot and dry; tongue dry and tremulous; one copious stool, natural; pulse 130 to 136; takes now about ten grains of quinine, in one or two grain doses, daily, and of brandy half an ounce every two hours; also, morphine in small doses.

April 13. Appetite returned; tympanitis much diminished; tongue moist and clearer; skin quite comfortable; looks better; pulse 130 to 136. Stimulants and nourishment constantly kept up, quinine as before and morphine one-sixteenth of a grain every three hours.

April 14. Although extremely weak and prostrated, her condition seemed more favorable than it had been; the pulse ranged in the neighborhood of 130, and was feeble and irritable; bowels rather too open; the tympanitis entirely gone; she complained of some sore throat, and coughed occasionally; morphine was now exchanged for pulv. opii, ten grains daily, and port wine was given as well as brandy. From the 14th to the 26th, her strength has very gradually increased, though not yet able to sit up alone in bed; the pulse has changed but little, one day being at 116, the next at 140; her cough had grown much more frequent and troublesome, attended with some muco-purulent expectoration; about the 16th, physical examination gave a crackling over the whole chest and the commencement of pulmonary abscesses was thought probable. She has had night sweats most of the time, though they have not always been profuse. The bowels have sometimes been quite free, the stomach has become able to digest farina, arrowroot, etc., the morphine has been gradually withdrawn and quinine and brandy have been freely given.

April 29, 5, P. M. Pulse 136; respiration 26; cough very troublesome, but without expectoration; bowels rather free; tongue moist and coated in patches; lips and hands tremulous, she is more feeble than usual; continued to take twelve grains of quinine and six ounces of brandy, and eight ounces of port, daily.

9, P. M. Pulse too rapid and feeble to be counted; respiration 48; half an hour ago, she complained to one of the patients of pain in right side near the region of the liver; is found moaning and sighing much; has a sharp pain on deep inspiration, and much tenderness on pressure over a considerable portion of right hypochondrium; no vomiting; no tympanitis.

11, P. M. Pulse 160; respiration 48; seems easier; coughs constantly.

April 30, 6, P. M. Pulse 160, or over; respiration 36.

12, M. Began to take one-quarter grain morphia every second hour, which made her apparently more comfortable.

From this time till her death, which occurred May 3, the sweats continued; the pulse was sometimes 160, sometimes 140; the respiration at times 36 and 40, at other times 28; the tongue was oftener dry than moist, and her little remaining strength gradually failed. On the first of May, she began to complain of some pain in the right side, and there was some tympanitis; on the second and third she kept her thighs flexed on the trunk, and the tympanitis increased. She became somewhat delirious, with frequent moaning and sighing, and she passed into incomplete coma, in which state she died.

*Post-mortem twenty-five hours after death. External appearances*

Moderate tympanitis, no erythematous spots; discharge from vagina sanious; rigor mortis as usual.

*Abdomen.* Moderately inflated with gas; no vascular congestion, except in dependent portions of large and small intestines; no serum; no pus; no recent lymph; no adhesions except of colon to liver, and of duodenum to left lobe of liver. These adhesions were as firm as the original tissues, consequently more than a month old; therefore, no evidence of peritonitis during late sickness; no evidence of inflammation in right iliac region; solitary glands of duodenum slightly elevated; mucous membrane somewhat vascular; glands of ilium very distinct and opaque; Peyer's patches not visible.

*Lungs.* Bronchial tubes red; considerably congested, containing bloody mucus; bronchial glands enlarged; posterior portion of each lung œdematous, a few military tubercles in right lung; moderate sized masses in left, more abundant than in right; lungs softened; no secondary abscesses discovered.

*Stomach.* Mucous membrane rather opaque; numerous ecchymotic spots on cardiac portion, and some over pyloric and mammillated.

*Liver.* Soft, of natural color; several whitish spots, of small size, upon its surface; veins open and apparently healthy, yellowish spots about the size of the lobules of Kiernan, and somewhat seen in the interior.

*Spleen.* Not enlarged; rather soft.

*Kidney.* Rather small; vascular, but apparently healthy; in pelvis large veins filled with fluid blood, lining membrane not blood-stained.

*Uterus.* Reduced to three inches in length by two inches and a half in width; walls thin and looking somewhat granular; inner surface covered with bloody exudation, of dark hue, outside of which was a grayish granular fibrinous deposit; a little milky muco-purulent matter in right fallopian tube.

*Fallopian Tubes.* Congested.

*Ovary.* Several large graaffian vesicles, containing fluid, opaque.

*Brain.* Vessels of dura mater somewhat injected, of pia mater, moderately; arachnoid raised from brain by a considerable effusion of transparent serum; also dotted with several irregular opaque spots; convolutions prominent and deepened and widened at vertex; several deep indentations made by the effused serum; a little serum in the ventricle, otherwise healthy; about four ounces of serum in the cavity of the brain.

No pus found in the knee-joints; nor any appearance of such deposit in other joints; posterior to right breast a small collection of purulent matter.

*Heart.* Of natural size, valves healthy, lining membranes not blood-stained, contains no blood.

The striking features of this case, is, that after the subsidence of the tenderness and tympanitis, on the 12th and 13th of April, there was no corresponding diminution of the frequency of the pulse, and but a limited improvement of strength. Her appetite was but imperfectly restored, and yet, she ate enough to sustain life till the last day or two. Her sweating was extraordinary; for two weeks past, there has hardly been a day when at the hour of the attending physician's visit she had not been sweating profusely, and that visit has been made at times, varying from half-past 9, A. M., to 5, P. M. She always spoke of herself as doing well, "first-rate," when it seemed as if she must have been exhausted by the perspiration. Her tongue during the last fortnight, though sometimes moist, has been commonly dry, and frequently there have been sordes on the teeth and lips. Every day or two there was a recurrence of the green vomiting. The vaginal discharge it was reported had ceased, but when searched on the day before her death, it was found that there was still a little pus at the vulva. That the pulse did not improve with the other symptoms, was constantly forbidding the hope of recovery.

For the last three days of life, there has been some increase in the frequency of the pulse. For three weeks past, it has been the conviction that the patient was suffering from some secondary purulent deposit, and it has been an object of almost daily search, to discover its seat. When the cough began, the attending physician was prepared to see the evidence of it in the lungs, but the expectoration of purulent matter was never abundant enough to confirm such a conjecture. When there was tenderness over the liver, it occurred to him that that organ might have become its seat, but there were no inequalities on its surface, and there was no jaundice. The *autopsy* itself failed to demonstrate any considerable deposit of this product; none could be found in the cavity of the abdomen, none in the lungs or brain, or gluteal region, or knee-joints, or either iliac fossa, or pelvis, or in the kidneys. In the liver were certain yellowish-white spots as large as the lobules of Kiernan, or larger, reserved for microscopical examination. Suspicious looking glands were noticed, anterior to and between the great abdominal vessels, and in the extremities of the broad ligament, also, one or two between the bladder and uterus; and there was a small collection of pus behind the right breast. Beyond this there was no pus discovered by the unaided eye, after diligent search. There was universal bronchitis, to account for the cough. The solitary glands in the duodenum, and in part of the ileum, were very large and apparently filled with pus, while the mucous membrane of these parts was thickened, and that of the duodenum very irregularly, but vividly vascular. This would probably account for the diarrhœa. The brain, or rather meninges were the seat of secondary inflammation, to explain the concluding symptoms.

But to account for the frequent pulse for thirty consecutive days, there was nothing but a fluid state of the blood, and the suspicion that it was contaminated by pus. The uterus still contained the evidence of an exhausted endo-metritis, and of little other disease. The peritoneum was free from all marks of inflammatory disease, either recent, or within a month of death. The mucous membrane of the stomach was thickened; the pyloric portion mammillated, and all the other portions thick-set with punctate ecchymosis. Here was probably the explanation of the vomiting.

The foregoing cases are those which had a fatal termination. Of the seven, three presented the lesions of peritonitis, viz., Nos. 2, 3, and 4. None of these were subjected to the systematic opium treatment. No. 2 was not seen by me during life. No. 3 came under my charge after the disease had made insidious progress for four days, having apparently yielded at first to a few half-grain doses of morphia. No. 4 was a rapid case, and ran its course during my temporary absence from town. I witnessed only the autopsy. The other four, it can hardly be doubted, were cases of purulent infection. The three following are cases of the same nature, unless Case No. 10 should be regarded as one of doubtful diagnosis. These three recovered.

Case VIII. Margaret B.; labor of twenty-one hours' duration; delivered, April 3, 1857.

April 10. Vaginal discharge became scanty, and somewhat fœtid.

12th. S me diarrhœa; secretion of milk, till now copious, greatly diminished; skin hot; countenance anxious; no chill; no abdominal pain or tenderness; from delivery has not gained strength, and pulse frequently above 100; now 120 and upward; perspiration at night.

13th. Green vomiting very troublesome; sweatiug in the morning; pulse 112 to 68, under influence of medicine.

14th. Sweats again in the evening; tympanitis; pulse 88 to 116.

15th. Tympanitis increased; pulse at or near 100; vaginal discharge light yellow.

16th. Pulse 104 to 81; average lower than yesterday; vaginal discharge ceased; appetite and general appearance improving.

17th. Pulse reduced, 96 to 76; still vomits; tympanitis continues; general condition better.

18th. Pulse variable, 96 to 76; vaginal discharge returned, and of dark color; tympanitis diminshing; no tenderness.

19th, 20th, and 21st. Still improving.

24th. Bowels moved for the first time spontaneously.

26th. Sitting up; feels well; appetite good; abscess of the breast began during convalescence; discharged, cured, May 22.

*Treatment.* Tinct. veratrum viride and sulph. morphiæ together, from the 12th to the 17th; after that the morphia alone.

Case IX. Mary S.; labor natural; duration, five hours and three-quarters; delivered, April 6; chill followed by tenderness in the hypogastrium; frequent pulse, hot skin, and restlessness twenty-four hours after.

8th. Pulse 134; pain and tenderness in hypogastrium.

9th. Under the influence of tinct. of veratrum viride and morphia; pulse fell to 84, even to 60, 58, and 56; still tenderness marked; spontaneous stools; vomited frequently; some mammary secretion; considerable pain over whole abdomen, and in the breasts.

10th. Symptoms nearly the same; vomiting has become green; pulse 58 to 96.

11th. Green vomiting; somewhat delirious; tenderness over uterus, and in right iliac region, has increased; tympanitis; free perspirations.

12th. Tympanitis increased; vaginal secretion scanty; tympanitis diminished, and uterus can now be felt, very large and tender; pressure on it produces hiccough, as also, on the whole abdomen; perspirations; some appetite; pulse 58 to 78; at night, severe pain in hypogastric region, and in direction of right ovary; tendency to hiccough.

13th. Some pain and tenderness; perspirations; restless; vomits; still some appetite; pulse the same; evening, profuse sweats.

14th. Perspirations more profuse; mammary secretion scanty; vaginal discharge, light yellow.

15th, 16th, 17th and 18th. Pain and tenderness had diminished; pulse 80 to 104.

18th. The mammary secretion returning; the last three days, profuse and nearly uninterrupted sweat; vaginal discharge still light yellow.

19th, 20th, and 21st. Improving in strength and appetite; still sweating profusely, day and night.

21st. Hæmorrhage from uterus.

22d, 23d, and 24th. Still these extraordinary sweatings, almost without interruption; takes quinine and brandy, and improves daily.

26th. No sweating; uterine tumor much diminished; feels well; appetite good; sat up for the first time; eight or ten days later, was discharged cured (May 4.)

*Treatment.* Tinct. of veratrum viride and sol. sulph. morphiæ; after the sweats commenced, sulph of quinia, brandy, and food. The veratrum viride controlled the frequency of the pulse with great certainty, but had no influence over the pain. This was subdued by morphine.

Case X. Julia K.; labor, very protracted, second stage lasting sixty-two hours; delivered, April 11, 1857.

April 12. Toward morning, had a slight chill; at 10, P. M., pulse 140; skin hot; tympanitis; no abdominal pain or tenderness.

13th. Vomiting of acid, colorless fluid; bowels moved by castor oil; pulse 132 to 112.

14th. Pulse 116 to 108; skin, for most part, cool and dry.

15th. Vaginal discharge, light yellow; skin dry; at 9, P. M, slight perspiration, for the first time, pulse going up from 96 to 116.

16th. Pulse 112 to 88; mammary secretion scanty, and has been.

17th. Pulse 92 to 80; tympanitis and vaginal discharge the same.

18th. Tympanitis diminishing; pulse 88 to 64.

19th, 20th, and 21st. Pulse, average, about 70; improving.

22d. Bowels moved, for the first time, spontaneously.

26th. Sits up; abscess of right breast beginning, April 26, attended by free suppuration; discharged, cured, May 4.

*Treatment.* Sol. sulph. morphiæ, and, for the first twenty-four hours, the tinct. of veratrum viride.

Another fatal case is to be added to this list, but as a *post-mortem* examination could not be obtained, and the lesions, therefore, cannot certainly be known, it is thought better to separate it from both the classes here recognized, and present it alone.

Case XI. Catharine F., delivered, April 1, at half-past 8, P. M., of a still-born child, after a natural labor of about twelve hours' duration; two days after, very nervous and excitable, but made little complaint; pulse about 130; camphor and Dover powder were administered with some relief.

No notes have been preserved of this case, but it is remembered that, from the 3d to the 10th, she had at first constipation of the bowels, and that this was followed by a profuse and troublesome diarrhœa; that she grew weak pretty rapidly; that the pulse continued very frequent; that she sweated freely, and did not sleep at night; that she did not complain of pain in the bowels, and had little or no tenderness; that she had pain in the position of the breasts; that it was found that the right breast was congenitally deficient; and that there was only moderate tympanitis; in the latter part of this period, the sweats were profuse, and she vomited freely (character of vomit not remembered); on the evening of the fourth, she was transferred to the fever wards; pulse then 160; extreme irritability; great tympanitis; grew weaker during the night, and died on the morning of the 12th; no autopsy allowed.

The history of these cases will not be complete till certain facts relating to their mode of occurrence, and their association one with another, are recited. During the months of January, February, and March, the physicians attending the lying-in patients, often spoke of the frequent occurrence of abscess in and about the pelvis, after labor. A death from metro-peritonitis is recorded in the hospital books, occurring on February 26; another on March 11; and on this latter day a death from peritonitis, all in persons recently delivered. The number of women delivered in the institution, during the month of February, was twenty-nine; during March, twenty-five.

Nothing was noticed in the lying-in wards to excite apprehension, from March 11 to April 2 or 3; from that date, three of the cases here narrated, took their origin, viz., No. 2, a case of metro-peritoneal puerperal fever; No. 11, probably of the some character; and No. 7, one of purulent infection, it will hardly be doubted, without peritonitis. Of these, one was delivered on March 27, and the other two on April 1 and 2. Nine women were delivered between March 27 and April 1; three of whom fell sick, and two of whom had well-marked peritonitis, and the other was supposed to have had the same affection, (Nos. 2, 3, and 11,) all of whom died. From the 2d to the 7th, inclusive, five women were delivered, four of whom fell sick, all with purulent infection and without peritonitis, (Nos. 6, 7, 8, and 9,) of whom two died and two recovered. From the 8th to the 12th, five were delivered two of whom took the sickness, (Nos. 5 and 10,) of these one recovered, the other died; neither had peritonitis.

On the 12th, the women waiting to be confined were all removed from the lying-in wards; new wards were opened in a distant part of the building, and the physicians, nurses, and furniture were entirely changed; *women who had been waiting in the lying-in wards were not admitted into the new wards*, but were sent to other wards in the same wing; there were eight in number. The lying-in wards were soon completely emptied; Dr. Lee, and his assistant, Dr. Buist, who had the charge of the lying-in wards thus far, still retained the care of the eight sent to other wards in the wing. These eight were delivered one after another, during the next three weeks, and one only took the disease, (No. 4); she was delivered, April 21, and attacked the next day; she had puerperal peritonitis, and died.

The newly-opened lying-in wards were filled by women who had been waiting in a building detached from the hospital proper, and who had had no intercourse with the old lying-in wards; these and the newly-received patients were sent to the new wards. From April 13 to May 1, ten women were delivered in these wards, all of whom did well, except Clara Byrne, (Case 1); she was delivered on April 26; the young physician then in charge of the wards had seen none of the cases that had previously occurred, and as has been said, the nurses and furniture, as well as the physicians, were all new and uncontaminated. On May 2, one of the house physicians, who up to ten days before had studied the previous cases, and had attended most of the *post-mortem* examinations, took charge of these wards. During these ten days, he had carefully avoided all sources of contamination, to fit himself for his turn of obstetrical duty, which now commenced; whether this change of physicians had any bearing on the development of the disease in this instance, must be betermined, if determined at all, by an examination of the case as it is recorded. It is pertinent to add that, during the month of May, this physician delivered twenty-three women in these wards, none of whom had fully developed puerperal fever, in either of its forms: while several had, for many days, very threatening symptoms, which excited his fears and those of the attending physician that the disease was not wholly eradicated.

Whether this form of phlebitis should be considered a form of puerperal fever may admit of discussion, but it certainly appeared in an epidemic form, as puerperal fever often appears; it spread, as puerperal fever spreads under like circumstances; and was checked by the same means that are relied on in hospitals for checking puerperal fever; while it occurred in the midst of, and at the same time with, cases, which no physician will hesitate to call puerperal peritonitis, if not puerperal fever.

One case more will complete this branch of the subject, and it is offered to show that this form of phlebitis is not confined to hospital practice, and may be sporadic. A young woman gave birth to a child at a station-house, May 9, 1857. On the the 12th, she was admitted to the Bellevue Hospital, sick; she had tenderness over the uterus, and some tympanitis; the vaginal discharge was abundant, dark, bloody, and very offensive; tongue furred, but moist; bowels confined; no mammary secretion; pulse 110. There was little change in her condition for one week, when the tympanitis subsided, and the tenderness over the uterus diminished; while the lochia became less offensive, and more natural, and there was some milk. She had, however, had moderate perspirations for the last few days; from about the 17th to the 25th, the sweats formed the prominent feature of her disease, they were profuse and prolonged, and frequently repeated. Her pulse was, however, but little excited, and her countenance and spirits were good; after this, the sweats diminished in severity and frequency, but on the 3d of June, an abscess commenced in the right breast, which was opened on the 8th, discharging a pint of fœtid pus. It is still under treatment by pressure, (June 17,) discharging from ℥i. to ℥ss. of pus daily; the woman's general health is nearly restored, she had profuse night-sweats from the 8th to the 12th, and these have now subsided.

Thus, of the four cases that survived this phlebitis, three had, during convalescence, abscess of the breast; and the exceptional case was discharged from the hospital before the abscess occurred in two who were attacked about the same time with her.

Fatal cases of this endo-metritis and puerperal phlebitis, (it may receive the last of these names, even if it is not admitted among the forms of puerperal fever,) came under my notice during the last winter, in private practice.

[Dr. Clark presented a tabular arrangement of the symptoms in nine cases of puerperal fever, attended by peritonitis, and also in five cases without peritoneal inflammation. His remarks on this branch of the subject, will be given in our next number.]

Prof. Barker said: The subject is one of great importance to every obstetrician, and in this country every practitioner is an obstetrician. The subject as announced is, "the contagiousness and treatment of puerperal fever," and the question must turn on the pathology. What is its essential character? Is it a local phlegmasia; or, is it a zymotic disease? I had the pleasure of being present the first evening, but being unfortunately absent the second evening, I have read with great care, the remarks of Prof. Clark, as reported in the N. Y. Journal of Medicine. I have been deeply interested in his remarks this evening, and feel greatly indebted for his researches and contributions to the morbid anatomy of the disease in question. But has this disease an anatomical character? If I understand him, it has, consisting of four primary lesions, viz., inflammation of the peritoneum; inflammation of the veins of the body of the uterus; inflammation of the lymphatics of the uterus; inflammation of the inner surface of the uterus or endo-metritis, which he considers to be in fact a limited phlebitis.

Now here, with all due respect, I am compelled to differ from him, having been accustomed to regard these lesions as being the relation of an effect, instead of a cause. I have believed that there was no proportionate relation between the intensity of the symptoms and the amount of the local lesion; I have regarded puerperal fever as a distinct, essential disease, having associated with it most generally, lesions of the peritoneum, or of the veins of the uterus, etc., but I have also believed that we may have peritonitis, or phlebitis, or any other of the local inflammations, even in the puerperal woman and not have puerperal fever. For example, we meet with phlebitis in the parturient woman in three forms: the adhesive inflammation, as in the disease known as *phlegmasia alba dolens;* the circumscribed puriform inflammation, in which little abscesses are found in the substance of the uterus; and the diffuse puriform inflammation, constituting pyæmia. The very interesting case which Prof. Clark has so minutely detailed, if met with alone, seems to me to be one of pyæmia, rather than a case of puerperal fever.

(To be Continued.)

